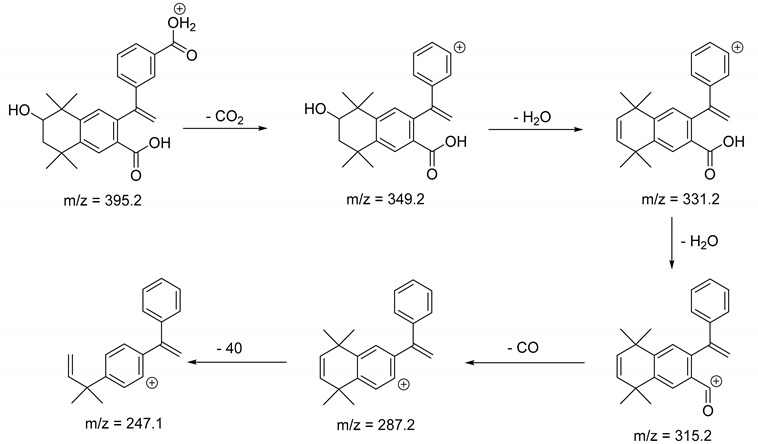# Photodegradation of Bexarotene and Its Implication for Cytotoxicity

**DOI:** 10.3390/pharmaceutics13081220

**Published:** 2021-08-07

**Authors:** Agata Kryczyk-Poprawa, István Zupkó, Péter Bérdi, Paweł Żmudzki, Joanna Piotrowska, Elżbieta Pękala, Aleksandra Berdys, Bożena Muszyńska, Włodzimierz Opoka

**Affiliations:** 1 Department of Inorganic and Analytical Chemistry, Jagiellonian University Medical College, 30-688 Kraków, Poland; joanna.piotrowska@uj.edu.pl (J.P.); wlodzimierz.opoka@uj.edu.pl (W.O.); 2 Department of Pharmacodynamics and Biopharmacy, University of Szeged, H-6720 Szeged, Hungary; zupko@pharm.u-szeged.hu (I.Z.); berdi.peter@pharm.u-szeged.hu (P.B.); 3 Interdisciplinary Centre for Natural Products, University of Szeged, H-6720 Szeged, Hungary; 4 Department of Medicinal Chemistry, Jagiellonian University Medical College, 30-688 Kraków, Poland; pawel.zmudzki@uj.edu.pl; 5 Department of Pharmaceutical Biochemistry, Jagiellonian University Medical College, 30-688 Kraków, Poland; elzbieta.pekala@uj.edu.pl; 6 Independent Researchers, 30-688 Kraków, Poland; oberdys20@gmail.com; 7 Department of Pharmaceutical Botany, Jagiellonian University Collegium Medicum, 30-688 Kraków, Poland; muchon@poczta.fm

**Keywords:** bexarotene, photostability, UV absorbers, third-generation retinoids

## Abstract

A detailed understanding of the stability of an active pharmaceutical ingredient and a pharmaceutical dosage form is essential for the drug-development process and for safe and effective use of medicines. Photostability testing as an inherent part of stability studies provides valuable knowledge on degradation pathways and structures of products generated under UV irradiation. Photostability is particularly important for topically administered drugs, as they are more exposed to UV radiation. Bexarotene is a more recent third-generation retinoid approved by the U.S. Food and Drug Administration and the European Medicines Agency as a topically applied anticancer agent. The present study aimed to assess bexarotene photostability, including the presence of UV filters, which have been permitted to be used in cosmetic products in Europe and the USA. The bexarotene photostability testing was performed in ethanol solutions and in formulations applied on PMMA plates. The UPLC-MS/MS technique was used to determine the tested substance. The presence of photocatalysts such as TiO_2_ or ZnO, as well as the organic UV filters avobenzone, benzophenone-3, meradimate, and homosalate, could contribute to degradation of bexarotene under UV irradiation. Four photocatalytic degradation products of bexarotene were identified for the first time. The antiproliferative properties of the degradation products of bexarotene were assessed by MTT assay on a panel of human adherent cancer cells, and concentration-dependent growth inhibition was evidenced on all tested cell lines. The cytotoxicity of the formed products after 4 h of UV irradiation was significantly higher than that of the parent compound (*p* < 0.05). Furthermore non-cancerous murine fibroblasts exhibited marked concentration-dependent inhibition by bexarotene, while the degradation products elicited more pronounced antiproliferative action only at the highest applied concentration.

## 1. Introduction

The development of pharmaceuticals aims to introduce new drug products in the market. This process has general requirements established by The International Council for Harmonisation of Technical Requirements for Pharmaceuticals for Human Use (ICH). ICH provides guidelines on quality, safety, efficacy, and multidisciplinary topics. The quality guidelines are divided into 14 categories (ICH Q1–Q14), and provide comprehensive recommendations and methodologies based on good manufacturing practices. The Q1A–Q1F stability documents address the stability testing of both new drug substances and products, and are related to the influence of humidity, temperature, pH, the presence of oxidizing agents, and light on their chemical and physical state. The annex to the main stability guideline is the Photostability Testing of New Drug Substances and Products (Q1B) implemented in Europe on 1 December 1996 and in the USA on 1 May 1997. This document states that light testing should constitute an integral part of stress testing of new drug substances and products [1]. The degradation products are potential impurities of drugs in addition to other organic (e.g., substrates from synthesis process, reagents, catalysts) and inorganic impurities (e.g., reagents, catalysts, heavy metals) [2,3].

Retinoids have become increasingly popular in recent years as an active ingredient of drugs and cosmetics. These drugs are intended for topical and systemic treatment of skin disorders including acne, psoriasis, and cancer, as well as an option to prevent and minimize signs of aging [4,5,6]. Three generations of retinoids are distinguished, with substantial differences in their structures. Recently, trifarotene, the first fourth-generation retinoid, has been reported for the treatment of acne vulgaris [7,8]. Because retinoids are commonly applied directly to the skin, their photostability should be thoroughly investigated [9]. Retinoids are a heterogeneous group of compounds in terms of photostability [10]. Most photostability studies have been conducted for first-generation retinoids, e.g., retinol, tretinoin, and isotretinoin. The results of these studies indicated that these compounds could undergo degradation under UV irradiation, which leads to the loss of activity, or can even cause photoreaction, phototoxicity, and photocarcinogenicity [11,12]. They have been found to undergo photoisomerization, photooxidation, and photodegradation [12]. This is due to the presence of conjugated double bonds in their molecules. Because the physicochemical properties of the first-generation retinoids are known, attempts have been made to improve their photostability while retaining their original activity. Further searches for the derivatives of known retinoids revealed that synthetic polyaromatic retinoids showed promising photostability. Third-generation retinoids include adapalene, tazarotene, and bexarotene. They were designed by cyclization of the polyene side chain, and thus gained a more rigid structure [13]. It is widely known that third-generation retinoids are more photostable than the first-generation ones. Nonetheless, because little is known about their potential photodegradation pathways and the structure of their degradation products, it would be useful to elucidate these phenomena. Degradation of active pharmaceutical ingredients is a highly complex process that is affected by different factors.

The stability of micronised bexarotene has been determined according to ICH storage conditions. Furthermore, photostability investigations confirmed the stability of bexarotene, but it should be protected from extreme heat and from excessive light exposure [14]. Because of the lack of detailed information on the photostability of a more recent third-generation retinoid—bexarotene—the present study aimed to assess the effect of UV irradiation on its stability. Bexarotene (Targretin) gel and Targretin capsules have been approved by the U.S. Food and Drug Administration and the European Medicines Agency as an anticancer agent for the treatment of cutaneous T-cell lymphoma [15]. Retinoids are a powerful class of compounds that are currently being tested for anticancer activity [16]. Recently, bexarotene has also been extensively studied for its application in the treatment of non-small-cell lung cancer—oral bexarotene in combination with cisplatin and vinorelbine [17]; advanced or metastatic non-small-cell lung cancer—comparison of carboplatin, paclitaxel, and bexarotene with carboplatin and paclitaxel [18]; metastatic breast cancer [19]; aerodigestive tract cancer—first clinical trial of combination of erlotinib with bexarotene [20]; and acute myeloid leukemia [21]. Bexarotene, as a selective agonist of retinoid X receptors (RXRs), plays a significant role in cell proliferation, differentiation, and apoptosis. Bexarotene has been proven to have neuroprotective potential in ischemic stroke [22] and traumatic brain injury [23], and in models of Parkinson’s disease **[24]** and Alzheimer’s disease [25]. Moreover, retinoid receptors are expressed in epithelial ovarian cancer and may indicate an poor prognosis, and for these reasons, retinoids may be used in the treatment of ovarian and breast cancers. Bexarotene has also been used experimentally to prevent breast cancer in women with genetic risk [26].

A significant and often neglected issue is the comprehensive study of the photostability of pharmaceuticals [27]. Although the photostability testing of pharmaceuticals covers their manufacturing process and storage, it is also necessary to test photostability to enable the effective administration of drugs [9]. An extremely valuable research subject for topical agents applied to the skin is to assess the influence of UV absorbers on their photostability in different UVA and UVB irradiation ranges. Chemical and physical UV absorbers act by blocking different wavelengths to protect the skin from harmful effects of sunlight. The U.S. Food and Drug Administration regulates sun-protection products as over-the-counter drugs. However, in Europe, they are considered to be cosmetics. The toxicological activity of the degradation products can be observed in many pharmaceutically active substances and medicinal products, which in some cases may influence the therapeutic effect [28]. Photodegradation products are impurities that can affect drug safety and quality [29]. Therefore, it is necessary to define their structures and amounts to assess the toxicity and risk related to their presence in pharmaceutical products. In recent years, the application of in vitro models in evaluating the toxicity is increasing [30]. Numerous toxicity tests, including toxic/genotoxic risks and phototoxicity, should be carried out to investigate the potential hazardous effects caused by the presence of degradation products.

Therefore, we performed a detailed investigation on the photostability of bexarotene in ethanol solutions and in prepared formulations applied on poly(methyl methacrylate) (PMMA) plates. Selected UV absorbers, which have been permitted to be used in cosmetic products in Europe and the USA, were used in the study to assess their impact on the stability of bexarotene under UV irradiation. The UPLC-MS/MS experiments were carried out for quantitative determinations of the investigated compound and identification of its photodegradation products. The in vitro assessment of the antiproliferative potential of the degradation products of bexarotene was performed using non-cancerous murine fibroblasts and human cancer cell lines isolated from breast (MDA-MB-231), cervix (HeLa), and ovarian (A2780) cancers.

## 2. Experimental

### 2.1. Materials and Methods

#### 2.1.1. Reagents

Bexarotene (4-[1-(3,5,5,8,8-pentamethyl-6,7-dihydronaphthalen-2-yl)ethenyl]benzoic acid) was purchased from Sigma-Aldrich (St. Louis, MO, USA). HPLC-grade methanol, acetonitrile, and formic acid (98%) were purchased from J.T. Baker. Ethanol (99.8%) was obtained from POCH (Gliwice, Poland). Water (quadruple-distilled) with a conductivity of less than 1 μS cm^−1^ was prepared using an S2-97A2 distillation apparatus (ChemLand, Stargard Szczecin, Poland). Zinc oxide (nanopowder, < 100 nm particle size) and titanium(IV) oxide (anatase, nanopowder, < 25 nm particle size, 99.7% (metal basis)) were purchased from Sigma-Aldrich. UV absorbers, namely avobenzone (AVB), 3-(4-methylbenzylidene) camphor (MBC), benzophenone-1 (BP-1), benzophenone-2 (BP-2), benzophenone-3 (BP-3), benzophenone-4 (BP-4), octocrylene (OC), homosalate, and meradimate, were obtained from Sigma-Aldrich. The formulation consisted of hydroxypropylcellulose (Klucel, Germany) and propylene glycol (VWR BDH Chemicals, France).

#### 2.1.2. Preparation of Reagent Solutions

A stock solution of bexarotene was prepared at a concentration of 0.2 mg mL^−1^ in a 50 mL volumetric flask by dissolving a weighted portion of the standard substance in ethanol, and the solution was then stored at 2–8 °C for no longer than 3 days. To validate the method, seven solutions with bexarotene concentrations of 0.01, 0.025, 0.05, 0.075, 0.1, 0.15, and 0.2 mg mL^−1^ were prepared.

#### 2.1.3. Bexarotene Gel Preparation (1 mg g^−1^)

A total of 20.475 g of anhydrous alcohol was weighed in a beaker, and 0.025 g of bexarotene was added. Subsequently, 3.75 g of propylene glycol and 0.75 g of hydroxypropylcellulose were added. The entire mixture was mixed using the T10 basic ULTRA-TURRAX^®^ homogenizer (IKA, Königswinter, Germany) at the lowest rotational speed (8000 rpm) for 40 min.

#### 2.1.4. Photostability Tests of Bexarotene in Ethanolic Solution

The irradiation of the bexarotene was conducted in ethanolic solutions with a concentration of 0.1 mg mL^−1^. The photodegradation experiments were performed in quartz Petri dishes with Ø of 50 mm (Hornik, Poland), covered with quartz covers to prevent any evaporation of ethanol, and secured with a parafilm. Next, 2 mL of the solution was placed on a quartz Petri dish and irradiated for 1 h at 500 W m^−2^ (cumulative dose of ultraviolet radiation: 218 kJ m^−2^) with a solar light simulator (Suntest CPSþ, Atlas, Germany) equipped with an optical filter for blocking wavelengths shorter than 290 nm and an IR-block filter to neutralize thermal effects.

Photocatalytic degradation experiments of bexarotene in ethanolic solution were performed with the addition of a TiO_2_/ZnO suspension in ethanol (50 mg TiO_2_/50 mg ZnO in a 5 mL flask). Briefly, 2 mL of solution containing 1 mL of bexarotene stock solution, 0.5 mL of ethanol, and 0.5 mL of TiO_2_/ZnO suspension was added to quartz Petri dishes and then exposed to irradiation. Analogously prepared samples covered with aluminum foil were used as dark controls. Samples were filtered through a 0.45 µm pore-size filter before the UPLC-MS/MS analysis. For chemical UV filters, 2 mL of solution containing 1 mL of stock solution of bexarotene, 0.2 mL of the investigated UV filter solution in ethanol, and 0.8 mL of ethanol was irradiated, and the samples were then analyzed by UPLC-MS. Three replicates of samples were prepared.

### 2.2. Photostability Tests of Bexarotene in Gel Formulation

#### 2.2.1. Preparation of Chemical Filters

First, 10 mg of a chemical filter was weighed in Eppendorf vials, which were filled with 1 g of propylene glycol. The entire mixture was mixed until complete dissolution was obtained.

#### 2.2.2. Preparation of Paste with Titanium(IV) Oxide and Zinc(II) Oxide at 5% Concentration

First, 5 g of paste containing 5% TiO_2_/ZnO (1:1, *w*/*w*) was prepared. Next, 0.125 g of each oxide was added to a mortar, followed by 4.75 g PEG 400. In addition, 5 g of paste with titanium(IV) oxide at 5% concentration was prepared. Then, 0.250 g TiO_2_ was weighed in a mortar, followed by the addition of 4.75 g of PEG 400.

#### 2.2.3. Preparation of Gels Containing Bexarotene and UV Filters

One gram of gel containing bexarotene and a 100 mg solution containing a UV filter were weighed. Both components were then mixed.

#### 2.2.4. Photostability Tests of Bexarotene in Gel Preparation

First, 50 mg of gel containing bexarotene and a UV filter was weighed onto each PMMA plate. The gel was evenly spread and left to dry for 10 min. The samples were then irradiated for 2 h. In addition, PMMA plates with gel containing only the active substance and with gel not containing the active substance and UV filter were prepared. After irradiation, the PMMA plates were rinsed with 2.5 mL ethanol. The samples were left alone for complete ethanol evaporation. The residues were dissolved in 0.5 mL ethanol, and the samples were then filtered through a syringe filter (nylon, 0.45 µm) prior to the UPLC-MS/MS analysis.

## 3. UPLC-MS/MS Analysis

The UPLC-MS/MS system comprised a Waters ACQUITY UPLC (Waters Corporation, Milford, MA, USA) coupled to a Waters TQD mass spectrometer (electrospray ionization mode: ESI-tandem quadrupole). Chromatographic separations were performed using an Acquity UPLC BEH (bridged ethyl hybrid) C18 column; 2.1 × 100 mm and 1.7 μm particle size, equipped with an Acquity UPLC BEH C18 VanGuard Pre-column; 2.1 × 5 mm and 1.7 μm particle size. The column was maintained at 40 °C and eluted under gradient conditions from 95% to 0% of eluent A over 10 min at a flow rate of 0.3 mL min^−1^ (eluent A: water/formic acid (0.1%, *v*/*v*); eluent B: acetonitrile/formic acid (0.1%, *v*/*v*)). Chromatograms were acquired using a Waters eλ PDA detector. According to the formula %i = (A_i_/(∑A) × 100), compound concentration (%i) after degradation of bexarotene was calculated as the quotient of the peak area (A_i_) to the sum of all peak areas (∑A) on chromatograms. Spectra were analyzed in the 200–700 nm range with 1.2 nm resolution and a sampling rate of 20 points s^−1^. MS detection settings for the Waters TQD mass spectrometer were as follows: source temperature, 150 °C; desolvation temperature, 350 °C; desolvation gas flow rate, 600 L h^−1^; cone gas flow, 100 L h^−1^; capillary potential, 3.00 kV; and cone potential, 30 V. Nitrogen was used as both the nebulizing and drying gas. The data were obtained in the scan mode ranging from 50 to 1000 m/z in time intervals of 0.5 s; eight scans were summed up to obtain the final spectrum. Collision-activated dissociation analyses were performed at an energy of 50 eV. Consequently, the ion spectra were obtained by scanning in the m/z 50 to 350 range. MassLynx V 4.1 software (Waters) was used for data acquisition.

## 4. Method Validation

The UPLC method was validated for the determination of bexarotene in the presence of photodegradation products in accordance with International Conference on Harmonisation, 2005. Validation of the method included an evaluation of the following parameters: specificity, linearity, limit of quantitation (LOQ), limit of detection (LOD), and precision.

## 5. In Vitro Cytotoxicity Assays

### 5.1. Investigated Solutions

The stock solution of bexarotene in ethanol was prepared in a 10 mL flask at a concentration of 10 Mm. The prepared samples, containing 2 mL of the above solution with the additional 10 mg of TiO_2_/ZnO (1;1, *w*/*w*), were placed into quartz dishes. The samples were irradiated for 1, 2, and 4 h in a solar light simulator. Later, the samples were filtered through a 0.45 μm filter before testing their antiproliferative properties.

### 5.2. Cell Lines and Cell Growth Inhibitory Assay

Human cancer cell lines isolated from breast (MDA-MB-231), cervix (HeLa), and ovarian (A2780) cancers were purchased from the European Collection of Cell Cultures (ECCAC, Salisbury, UK). Cells were cultivated in minimal essential medium supplemented with 10% fetal bovine serum, 1% antibiotic–antimycotic mixture, and non-essential amino acids All media and supplements were obtained from Lonza Group Ltd. (Basel, Switzerland). Near-confluent cells were seeded onto a 96-well microplate at a density of 5000 cells/well, and after overnight standing, new medium containing the tested substance dissolved in DMSO was added. After incubation for 72 h at 37 °C in humidified air containing 5% CO_2_, the viability of the cells was determined by the addition of 20 μL of MTT (3-(4,5-dimethylthiazol-2-yl)-2,5-diphenyltetrazolium bromide, 5 mg/mL) solution. After a 4 h contact period, the medium was removed, and the precipitated formazan crystals were dissolved in 100 μL of DMSO during a 60 min period of shaking. Finally, the produced formazan was assayed at 545 nm, using a microplate reader utilizing wells with untreated cells as control [25]. The highest concentration of DMSO (0.3%) exerted no substantial action on the growth of the utilized cells. All in vitro experiments were carried out on two microplates with at least five parallel wells.

### 5.3. In Silico Toxicity Prediction

The OSIRIS Property Explorer was used to predict mutagenicity, tumorigenicity, irritation, and reproductive effects of bexarotene and its photocatalytic degradation products [31].

## 6. Statistical Analysis

Statistical analyses were performed using Statistica v. 12 (StatSoft) and GraphPad Prism (GraphPad Software, San Diego, CA, USA). For statistical analysis of cell death, a two-way analysis of variance (ANOVA) with Tukey’s multiple-comparisons test was conducted. Differences were considered statistically significant in comparison with untreated controls when *p* ≤ 0.05.

## 7. Results and Discussion

Method Validation

**Figure 1:**
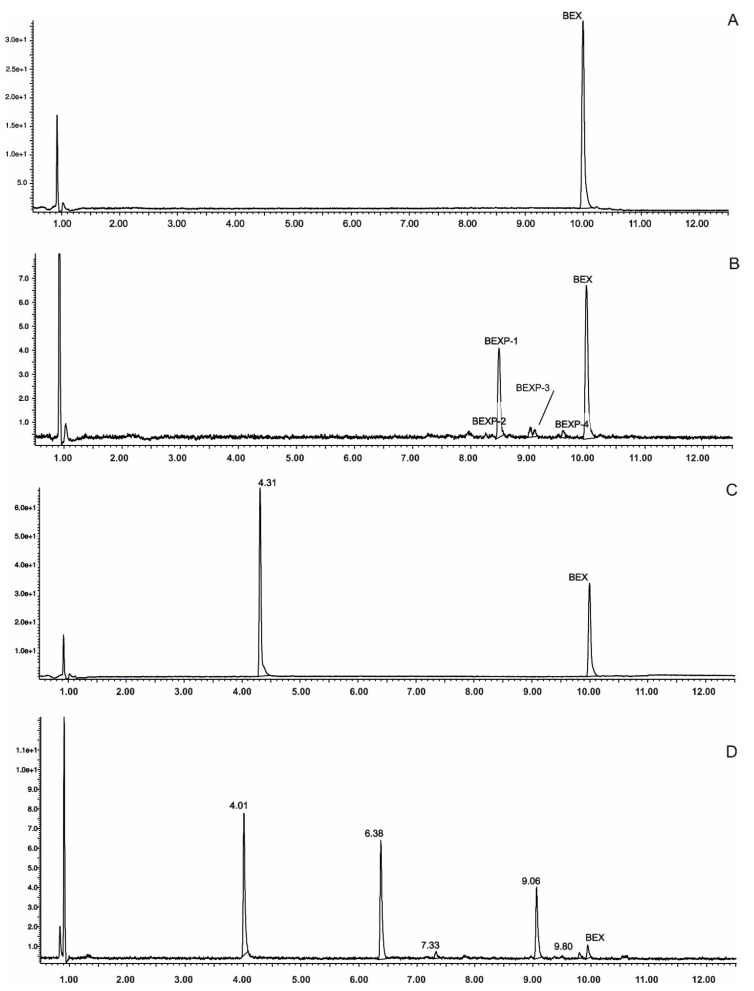
UPLC-MS/MS analysis of photostability of bexarotene: (**A**) pure standard substance; (**B**) photocatalytic degradation of bexarotene in ethanol solution after exposure to UV irradiation for 1 h in the presence of ZnO/TiO_2_; (**C**) bexarotene with BP-2 in ethanol solution after exposure to UV irradiation for 1 h; (**D**) bexarotene gel 1 mg g^−1^ with the addition of AVB after exposure to UV irradiation for 2 h.

The validation of the UPLC-MS/MS method confirmed its applicability for bexarotene photostability assessment. The developed method was specific to bexarotene and coexisting degradation products in the tested solutions, as well as in the prepared formulations. The specificity of the method was evaluated by the assessment of blank chromatograms, chromatograms of the pure standard substance, and chromatograms of bexarotene after degradation. Furthermore, the resolution of the investigated chemical UV absorbers and bexarotene was verified. Unfortunately, octocrylene and bexarotene were not well resolved; thus, the former compound was excluded from the subsequent study. The chromatographic separation of the other UV filters under study and bexarotene in ethanolic solutions was satisfactory, both before and after UV irradiation (Figure 1). In the next step, the separation of bexarotene and UV absorbers under study applied in the prepared formulations was verified to assess the potential interference with ingredients. The chromatograms of the samples of gel and gel with the addition of bexarotene and UV absorbers applied on PMMA plates were analyzed before and after their UV irradiation. No additional peaks were derived from gel ingredients. Least squares linear regression was applied to statistically evaluate the peak areas vs concentration of bexarotene relationship. The method linearity was determined for the concentration range of 0.01 mg/ mL^−1^ to 0.2 mg/ mL^−1^. The linear regression equation was y = 14555.1 × −67351. The correlation coefficient and determination coefficient (R^2^) obtained for the linear model were found to be 0.9987 and 0.9973, respectively. The *y*-intercept was statistically nonsignificant. The Shapiro–Wilk test was used to confirm normality assumption; the significance value (*p* = 0.2526) was >0.05, and hence, the residuals were considered to be normally distributed. On the basis of the standard error of residuals and the slope of the calibration plot, the calculated LOQ and LOD values were 0.01 and 0.04 mg mL^−1^, respectively. The precision was determined by the analysis of six replicates of bexarotene solutions at a single concentration of 0.1 mg mL^−1^ (%RSD = 0.83%).

## 8. Photostability Studies

### Identification of Degradation Photoproducts

The metabolism of bexarotene in patients with advanced skin lesions of cutaneous T-cell lymphoma was investigated following oral doses. Peak plasma concentrations were observed at 2 h after administration. Plasma protein binding of bexarotene is almost 99%, which has a significant impact on its distribution, elimination, and pharmacological effects [32,33]. After topical administration of bexarotene, a very low serum level (< 5 ng mL^−1^) was observed [33]. Bexarotene is primarily metabolized in the liver. Both 6- and 7-hydroxy-bexarotene and 6- and 7-oxo-bexarotene are the major metabolites of bexarotene that have been identified in plasma. The major cytochrome responsible for the formation of the oxidative metabolites of bexarotene is cytochrome P450 3A4 [34]. The oxidative metabolites are active in in vitro assays of retinoid receptor activation [35]. Photostability studies could provide valuable information about unwanted compounds that can develop during manufacturing, storage, and therapeutic use of drugs. The degradation products of bexarotene were identified by MS/MS analyses. The proposed structures of the degradation products are shown in Table 1, and the proposed fragmentation patterns are presented in Table 2. Bexarotene (4-[1-(3,5,5,8,8-pentamethyl-6,7-dihydronaphthalen-2-yl)ethenyl]benzoic acid) mainly underwent an oxidative degradation process. Photooxidation was caused by UV irradiation in the presence of the photocatalysts TiO_2_/ZnO. Under these conditions, hydroxyl radicals (HO·) were formed, which then underwent a radical substitution reaction [36,37]. Pathways of degradation of bexarotene involve hydroxylation of the tetrahydronaphthalene ring to 7-hydroxy-5,6,7,8-tetrahydronaphtyl (7-hydroxy-bexarotene—BEXP-4), which could lead to 7-oxo-5,6,7,8-tetrahydronaphtyl (7-oxo-bexarotene—BEXP-3) and ultimately could lead to the cleavage of the ring (BEXP-1). The other possibility is oxidation of the methyl group in position 3 of tetrahydronaphthalene ring to the corresponding carboxyl group (BEXP-1 and BEXP-4), or the formation of BEXP-2 (4-(3,5,5,8,8,-pentamethyl-5,6,7,8-tetrahydro-2-naphthyl) carbonyl) benzoic acid). BEXP-2 is used as a substrate to synthesize bexarotene with Grignard reagent in a solvent (e.g., diethyl ether, tetrahydrofuran, ethylene glycol, dimethyl ether) followed by dehydration using p-toluene sulfonic acid. BEXP-2 was also described as one of the two major impurities—identified as Impurity B in the patent: “Process for the preparation of highly pure bexarotene” [38].

## 9. UV Irradiation of Bexarotene in Solution in the Presence of UV Absorbers

In Europe, Annex VI to the EU Cosmetics Regulation contains 29 substances. The list includes only two physical filters: zinc(II) oxide and titanium(IV) oxide. They are accepted in both Europe and the USA. The benefit of these absorbers is a wide spectrum of UV protection, both against UVA and UVB. Zinc oxide has been extensively applied in cosmetics, including personal care products and pharmaceuticals. However, the high photocatalytic activity of TiO_2_ and ZnO could promote the degradation of the parent drug by generation of reactive oxygen species (ROS), mainly hydroxyl radicals (OH^•^) and oxygen radicals (O^•^), resulting in the oxidation of the organic compound [10,39].

**Figure 2:**
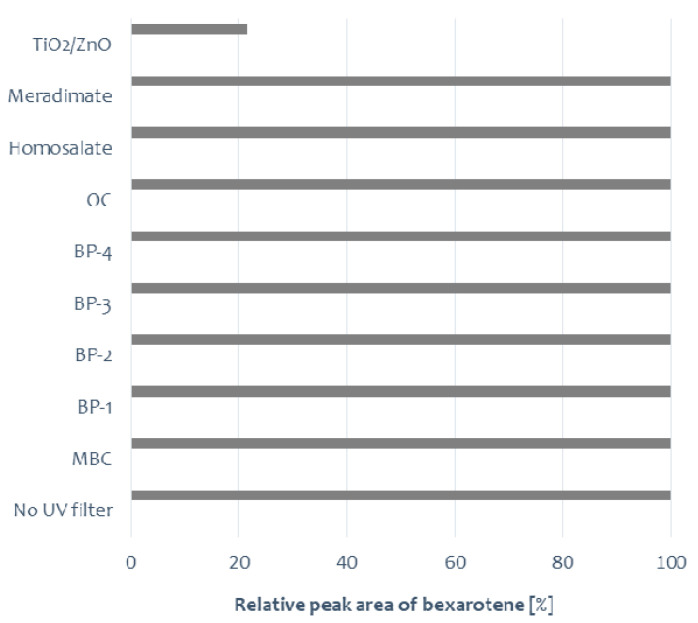
Average relative peak areas of bexarotene after 1 h of UV irradiation in the presence of UV absorbers.

The photostability testing of bexarotene was performed in solutions with and without the presence of UV absorbers. Irradiation was conducted for 1 h. Bexarotene degradation was observed only in the sample containing the ZnO/TiO_2_ mixture. In control samples containing bexarotene or bexarotene in the presence of UV filters, the tested substance remained stable (Figure 2). MBC isomerization was only observed (the additional peak on the chromatogram *m*/*z* = 255 corresponds to MBC). In the presence of ZnO/TiO_2_, a photocatalytic degradation of bexarotene was observed; several degradation products were recorded on chromatograms, and the probable structures of four of them are presented in Table 1.

**Figure 3:**
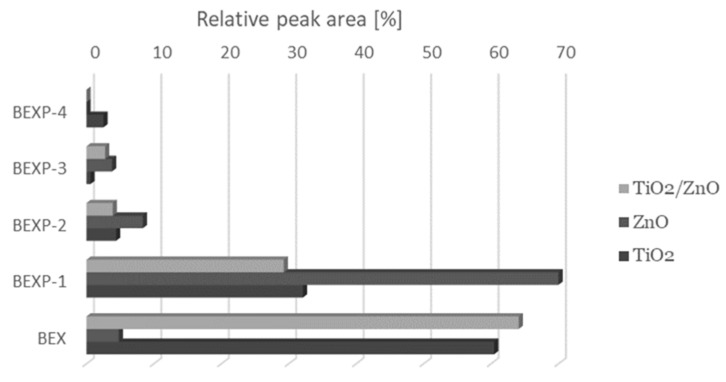
Average relative peak areas of the main photodegradation products of bexarotene after 30 min of UV irradiation evaluated with the UPLC-MS/MS technique.

In the subsequent study stage, we performed assessment of bexarotene photocatalytic degradation in the presence of TiO_2_, ZnO, or their mixture (ZnO/TiO_2_) used in equal amounts, 10 mg in 2 mL of the investigated solution. The prepared solutions were irradiated for 30 min. Dark control samples were also prepared. The investigated substance remained stable in control samples. Bexarotene degradation was observed in the applied conditions, and the type of catalyst applied influenced the quantitative and qualitative composition of the formed degradation products (Figure 3). A considerably lower bexarotene degradation was observed with TiO_2_ and ZnO/TiO_2_ application than with ZnO application (*p* < 0.05).

## 10. UV Irradiation of Bexarotene in Formulations

**Figure 4:**
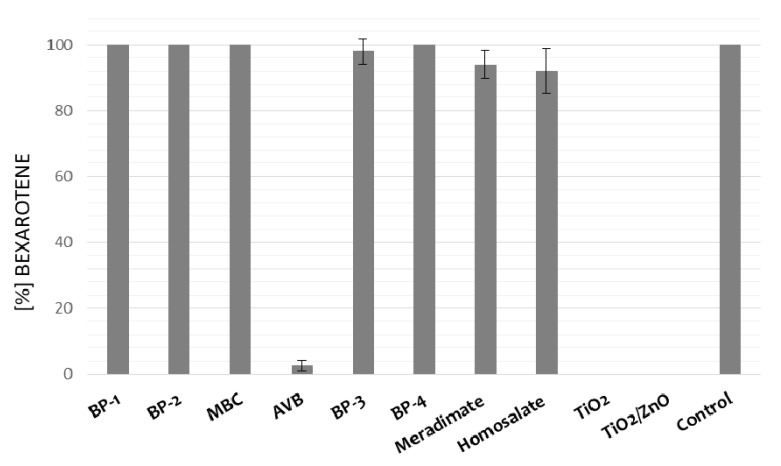
Percentage bexarotene content after 2 h UV irradiation of gel samples containing the investigated substance at 1 mg g^−1^ concentration and the tested chemical and physical filters.

Stability of the active substance contained in the drug form may differ from its stability in the powder or solution form; hence, it is valid to conduct stability testing of active pharmaceutical ingredients and finished pharmaceutical products. For a medicinal product, the presence of auxiliary substances may impact the kinetics of the degradation of the active substance. Photostability testing of bexarotene in the prepared formulations was conducted by irradiation of gel containing the investigated substance and gel containing bexarotene in the presence of selected chemical and physical UV absorbers. For our study, the prepared bexarotene gel with 1 mg g^−1^ was used. The prepared 50 mg samples were irradiated on PMMA plates for 2 h. In addition, dark control samples were also developed (the tested sample was wrapped in aluminum foil and irradiated for 2 h). The percentage of bexarotene content in the tested samples is shown in Figure 4.

The influence of the presence of a UV filter on bexarotene stability was assessed in experimental conditions. No changes in bexarotene concentration were observed after 2 h of irradiation with chemical filters: BP-1, BP-2, 4-MBC, and BP-4. Bexarotene underwent degradation in the presence of avobenzone or physical UV filters. The influence of formulation on the stability of the active substance should be emphasized here: bexarotene in gel in the presence of avobenzone underwent almost complete photodegradation, whereas in the same combination in ethanol solution, it remained stable (Figure 4). Moreover, it is well known that UV filters can exhibit photostabilizing effects, but may also contribute to accelerating degradation of active pharmaceutical ingredients under UV irradiation [36,39]. In the case of bexarotene, its photostability was affected by AVB, BP-3, meradimate, and homosalate.

### 10.1. In Vitro Antiproliferative Assays

**Figure 5:**
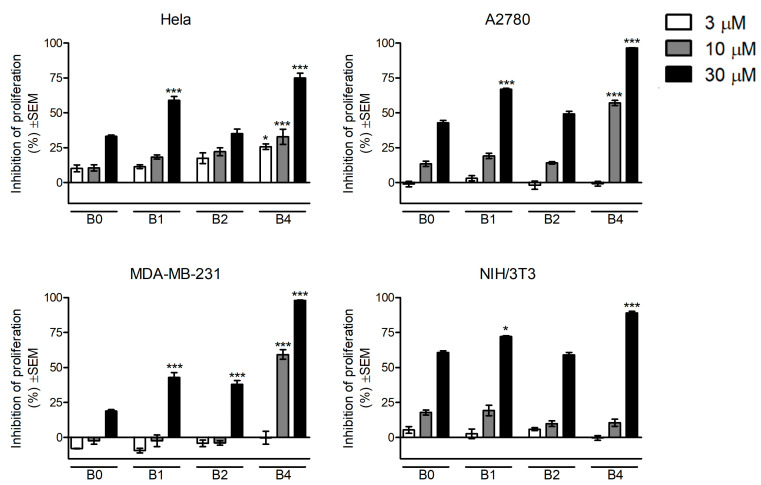
Changes in cell viability of cancer cells caused by the solutions of bexarotene following photocatalytic degradation experiments in HeLa, A2780, MDA-MB-231, and NIH/3T3 cells. Cells were treated with 3, 10, and 30 μM of bexarotene solutions after UV irradiation (B0—without irradiation, B1—1 h, B2—2 h, and B4—4 h of irradiation); * *p* < 0.05 and *** *p* < 0.001.

In vitro antiproliferative assays following photocatalytic degradation in experimental conditions were determined in vitro against cervical (HeLa), breast (MDA-MB-231), and ovarian cancer (A2780), as well as non-cancerous murine fibroblast (NIH/3T3) cell lines, by means of MTT assays. Cells were treated with 3, 10, and 30 μM of solution after UV irradiation (B0—without irradiation, B1—1 h, B2—2 h, and B4—4 h). The results are shown in Figure 5. The in vitro cytotoxicity risk evaluation based on photocatalytic degradation of bexarotene indicated substantial antiproliferative action at concentrations of 10 or 30 μM. HeLa cells seemed outstandingly sensitive, exhibiting significant inhibition of proliferation at even 3 μM of B4. Treatment of A2780 cells with bexarotene without irradiation resulted in pronounced growth inhibition at 30 μM, followed by a biphasic action in terms of the irradiation time. This biphasic character can be detected on HeLa cells as well, indicating that antiproliferative metabolites could be generated during the irradiation of bexarotene in a time-dependent way, and the produced metabolites are different in terms of cell-growth-inhibiting properties. Non-cancerous murine fibroblasts (NIH/3T3) exhibited marked concentration-dependent inhibition by bexarotene, while the degradation products elicited a more pronounced antiproliferative action only at the highest applied concentration.

### 10.2. In Silico Toxicity Predictions

The toxicities of bexarotene and all its identified photodegradation products were assessed with the OSIRIS Property Explorer server. None of the degradation products turned out to be harmful in terms of the mutagenic, tumorigenic, or irritant proprieties. Bexarotene and BEXP-4 demonstrated a positive risk assessment in terms of reproductive effects, which was related to the 1,1-diphenylethylene moiety.

## 11. Conclusions

The present article provides, for the first time, an insight into the photodegradation pathways of bexarotene, and reports the possible degradation products of this substance. In line with the ICH guidelines, photostability testing constitutes an integral part of investigating the stability of medicinal substances and pharmaceutical products. To assess bexarotene’s photostability, a new UPLC method was developed to determine the investigated compound in the presence of its degradation products. Due to its polyaromatic structure, bexarotene remained stable under the experimental conditions in ethanol solution after UV irradiation, except in the presence of TiO_2_ and ZnO. No changes in bexarotene concentration in the gel formulation were observed after irradiation with the chemical filters BP-1, BP-2, 4-MBC, and BP-4, but it underwent degradation in the presence of avobenzone or physical UV filters. This study revealed the impact of the presence of UV absorbers on the photostability of bexarotene in formulation. Non-cancerous murine fibroblasts showed a marked concentration-dependent inhibition by bexarotene, while the solution after photodegradation of bexarotene elicited a more pronounced antiproliferative effect only at the highest applied concentration.

## Figures and Tables

**Table 1:** Proposed structures of degradation products of bexarotene.

Compound	RT [min]	[M+H^+^]	Fragmentation Ions	Structure
BEXP-1	8.49	425.1 [M-H]^−^ 409.1 [M+H-H_2_O]^+^	ESI(−): 304.8, 348.01 ESI(+): 218.1, 293.1, 305.2, 323.2, 339.2, 365.1, 381.1	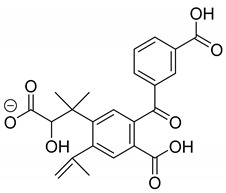
BEXP-2	9.03	351.2	149.0, 229.2, 239.1, 253.1, 267.1, 281.1, 297.1	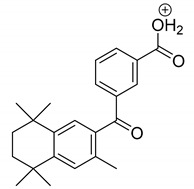
BEXP-3	9.11	365.1	149.0, 239.1, 253.1, 267.1, 281.1, 297.1	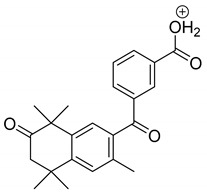
BEXP-4	9.48	395.2	247.1, 287.2, 315.2, 331.2, 349.2	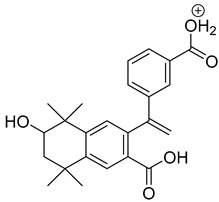
BEX	9.99	349.2	142.0, 227.2, 237.1, 251.1, 265.1, 279.1, 295.2	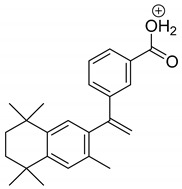

**Table 2:** Proposed fragmentation patterns.

Bexarotene
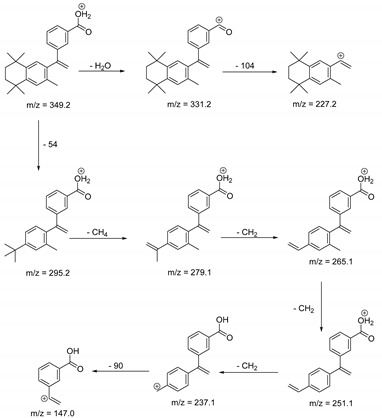
**BEXP-1** 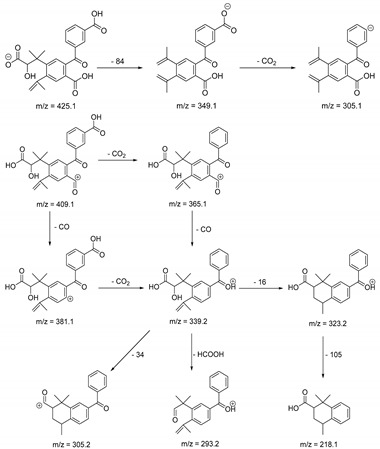
**BEXP-2 and BEXP-3** 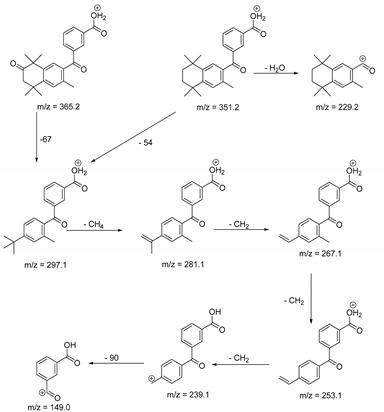
**BEXP-4**